# Polyethylene terephthalate microplastics exposure enhances the risk of ulcerative colitis: Insights from multi-omics integration, machine learning, and molecular docking reveal intestinal toxicity mechanisms: a commentary

**DOI:** 10.1097/JS9.0000000000004675

**Published:** 2026-01-07

**Authors:** Guosheng Lin, Fangfeng Lin, JinHong Liu

**Affiliations:** a Fujian University of Traditional Chinese Medicine, Fuzhou, China; b The Third Affiliated People’s Hospital of Fujian University of Traditional Chinese Medicine, Fuzhou, China; c The Affiliated People’s Hospital of Fujian University of Traditional Chinese Medicine, Fuzhou, China

*Dear Editor*,

We read with great interest the recent article entitled “Polyethylene Terephthalate Microplastics Exposure Enhances the Risk of Ulcerative Colitis” by Yang *et al*^[1]^. The authors are to be commended for integrating public transcriptomic datasets, machine learning, single-cell analysis, molecular docking, and *in vivo* validation to investigate how polyethylene terephthalate microplastics (PET-MPs) may contribute to the development or exacerbation of ulcerative colitis (UC). Their work provides an important foundation for understanding the potential molecular mechanisms linking environmental microplastic exposure with intestinal inflammation. While the study is comprehensive and forward-looking, we would like to raise several methodological considerations that we believe may further strengthen this promising line of research. This commentary complies with the “2025 TITAN Guidelines” for statements and use in managing artificial intelligence^[2]^. No artificial intelligence tools were used during the writing of this work.

First, the study identifies several potential PET-MPs–related targets through network toxicology and validates key genes involved in UC progression. However, the biological process by which PET-MPs enter intestinal epithelial or stromal cells and subsequently influence intracellular pathways remains insufficiently understood. If future work could integrate transcriptomic changes from PET-exposed animal colon tissues with human UC datasets, it may help refine and prioritize disease-relevant targets, thereby enhancing the credibility and translational value of the identified core genes.

Second, molecular docking was employed to explore the interactions between PET-MPs and the predicted protein targets. Although docking provides a valuable first step in assessing potential ligand–target affinity, PET-MPs are not conventional small molecules, and their physicochemical properties may not be fully captured by single-frame docking approaches. Incorporating all-atom molecular dynamics simulations in subsequent studies – such as short-timescale stability assessments and post-docking free energy calculations (e.g. Molecular Mechanics/Poisson–Boltzmann Surface Area or Molecular Mechanics/Generalized Born Surface Area) – may offer a more realistic representation of the conformational behavior and interaction stability between PET fragments and key UC-related proteins, as these end-state methods have been widely used to improve the reliability of protein–ligand binding predictions^[3,4]^.

Third, UC is a highly heterogeneous disease with substantial molecular differences between active and remission phases^[5]^. Therefore, it would be informative to examine the expression patterns of the identified PET-MPs–related targets across disease stages using independent datasets. Validating whether these core genes show consistent differential regulation in active versus quiescent UC could both enrich the biological interpretation and clarify whether PET-MPs preferentially influence particular inflammatory states. Prior transcriptomic studies have demonstrated distinct signatures between active and remissive UC samples, suggesting that stage-specific validation could materially affect target prioritization and mechanistic inference^[6]^.

In summary, this study provides valuable insights into the potential role of PET-MPs in modulating UC-related molecular pathways. Our comments are intended as constructive suggestions that may help refine mechanistic understanding and strengthen the interpretability of future PET-MPs toxicological studies. We applaud the authors for advancing this important and timely field of research and hope that these points will be useful for guiding subsequent investigations.

## Data Availability

Not applicable.
